# The Relation of Exophthalmic Goitre to Pregnancy and Obstetrics

**Published:** 1907-03-23

**Authors:** 


### THE RELATION OF EXOPHTHALMIC GOITRE TO PREGNANCY AND OBSTETRICS.

At the meeting of the Edinburgh Obstetrical Society on March 13 Sir Halliday Croom gave an account of his experience of exophthalmic goitre in its relation to gynæcology and obstetrics. After referring briefly to the nature of the disease, the speaker stated that though ten times as frequent in women as in men, it was comparatively rarely met with in obstetrical or gynæcological practice. This was chiefly noticeable in hospital work, as in the records of the Edinburgh Maternity Hospital, embracing over 15,000 cases, there was but one case of goitre referred to, and that of the ordinary type, and having no relation to Graves' disease. In the speaker's fifteen years' association with the gynæcological wards of the Royal Infirmary only two cases of exophthalmic goitre has been seen, both suffering from menstrual irregularity. The condition was more commonly met with in private practice, and its incidence in the speaker's cases ranged from fifteen to sixty-five years of age, the majority occurring about the thirty-fifth year. Women suffering from exophthalmic goitre usually suffer more or less discomfort during the menstrual period, and menstruation is usually profuse; but the condition is rarely associated with gross pelvic lesions. Only three cases requiring operation for pelvic lesions had been seen. (1) A middle-aged woman suffering from a fibroid tumour, who expired on the induction of anæsthesia before the operation was begun; (2) a single woman, aged 58, suffering from a large ovarian tumour and a uterine polypus, both conditions being successfully treated on separate occasions, but without alteration of the goitre; (3) a girl, aged 20, who suffered from monthly pain and had not menstruated, in whom an imperforate hymen was incised without change in the goitre. In the majority of cases irregular and profuse menstruation is the only pelvic symptom, but in older women ovarian atrophy may ensue with amenorrhœa. In two cases where exophthalmic goitre began about puberty the patients married and were sterile, with infantile pelvic organs. In a case of exophthalmic goitre, where the patient suffered from attacks of mania during the menstrual period, removal of both ovaries cured the mania, but did not benefit the Graves' disease.

As regards the influence of exophthalmic goitre on pregnancy the speaker had seen twelve cases, in eight of which the progress of gestation and labour was uneventful, but the latter seemed to aggravate the goitre, at least for a time. Of the other four cases, (1) suffered from severe post-partum hæmorrhage followed by superinvolution. (2) A lady in whom exophthalmic goitre had begun six months before the occurrence of her third pregnancy. She became much emaciated and suffered from hæmorrhage, for which labour was successfully induced. (3) A patient in whom abortion occurred at the third month of her first pregnancy, and was followed by profuse hæmorrhage and superinvolution, accompanied by decided improvement in the exophthalmic goitre. (4) A case in which convulsions occurred twelve hours after labour, and proved fatal in the course of the day. The influence of pregnancy on exophthalmic goitre is not only to favour its incidence, but apparently to aggravate the symptoms at least for a time—no case of cure of the Graves' disease by the occurrence of pregnancy having been seen.

The association of exophthalmic goitre with pelvic lesions is not common, and the treatment of the latter has no influence on the former. The effect of exophthalmic goitre on pregnancy does not appear sufficiently serious to warrant the prohibition of marriage to those suffering from that condition; or should they marry, the interdiction of pregnancy, but no hope of benefit from pregnancy should be encouraged, and the possibility of post-partum complications should be borne in mind.

